# Dermatology

**Published:** 1906-12

**Authors:** J. A. Nixon


### DERMATOLOGY.

**Effect of Diet on the Fat-secretion of the Skin.**—The investigation of the interdependence of the physiological processes of the skin and of the metabolism of the body as a whole is a subject which is receiving the attention of both dermatologists and pathological workers to a remarkable extent at the present time. A very valuable research in this province has recently been published by Rosenfeld, of Breslau,[1] which deals with the effect of diet on the fat-secretion of the skin. He starts with a résumé of the results obtained by other authorities, quoting notably those of Krukenberg and Leubuscher, who used a method which depended on the estimation of the fat excreted over a small area in a given time, from which the approximate amount could be calculated for the whole surface of the body—an estimation in which the errors became enormously magnified,

[1] Rosenfeld, *Zentralbl. f. innere Med.*, 1906, xxvii. 986

since the actual amount of fat collected was multiplied by 4,000 or 6,000 to give the total for the body. These two authorities arrived respectively at the conclusion that 40.8 gm. and 15 gm. per diem represented the whole fat-excretion of the skin. Rosenfeld, by a process that leaves little room for magnification of the experimental error, estimates the total fat at an amount varying, as will be seen, from 1 to 2.5 gm. per diem.

The subjects of his experiments (two students in his clinic) were clothed completely—body, head, hands and feet—in woollen underwear, which absorbed the whole of the sweat secreted. Upon removal the garments were soaked with and allowed to stand in chloroform for several days, then pressed in a powerful iron press, and the chloroform so extracted distilled, the residue dissolved in ether, filtered, and evaporated to dryness. The changes in the diet were as follows: During the first period Herr Reich, a spare man weighing 120 lb., height 179 cm., took per diem 250 gm. of meat, 20 gm. of cocoa, 94 gm. of egg, 70 gm. of fat (as butter), and carbohydrate 100 gm. of biscuit, and 332 gm. of sugar; during the second period 250 gm. of butter instead of 70, and 88 gm. of carbohydrate instead of 420. It was found that ordinary exercise, walking and fencing, did not affect the excretion, and that the health was equally good on either diet. With a diet rich in carbohydrates the amount of fat per diem was 2.3 gm.: on the fat diet this amount fell to 0.94 gm.

Herr Ludwig underwent the same test with similar results (*vide* table, Ludwig II.), and further, while taking the so-called carbohydrate diet, observed the effect of adding 90 gm. of absolute alcohol to his food. The amount of fat then excreted did not vary perceptibly, but the "iodine-figure" showed a notable decrease, proving a qualitative change had taken place in the fat. (*Vide* table, Ludwig I.)

For purposes of comparison two diabetic patients whose tolerance of carbohydrates was low (70 and 40 gm. of dextrose respectively) were submitted to an investigation of their ordinary fat-secretion on a diet containing these quantities of carbohydrate. Their fat-secretion was equal to that of the healthy subjects while on the "fat diet."

TABLE.

| Name. | Carbohydrates. | Fat. | Fat-secretion. | Iodine-figure. |
|---|---|---|---|---|
| | Gm. | Gm. | Gm. per diem. | |
| Reich—1st period ... ... ... | 420 | 100 | 2.3 | — |
| ,, 2nd ,, .. ... ... | 88 | 250 | 0.94 | — |
| Ludwig I.—1st period ... ... | 210 | 150 | 2.4 | 69.23 |
| ,, 2nd ,, ... ... | 210 | 150 | 2.3 | 58.32 |
| Ludwig II.—1st period ... | 390 | 100 | 2.2 | 70.84 |
| ,, 2nd ,, (alcohol) | 80 | 240 | 1.44 | 62.54 |
| V. (Diabetes) ... ... ... ... | 70 | — | 0.95 | — |
| H. (Diabetes) ... ... ... ... | 40 | — | 1.1 | — |

In diabetes with a diet free from carbohydrates and rich in fat there is little secretion of fat by the skin; at the same time the subjects show a tendency to develop boils and carbuncles. Rosenfeld propounds the question, "Is free fat-secretion a protection against boils?"

Ludwig in his experiment found that with an ordinary diet, rich in carbohydrates, he was able to take 4 gm. (60 grains) of potassium bromide per diem without any signs of bromide-acne. His fat-secretion was 2.05 gm.

Further questions suggested, but not experimentally answered, are, "Is a skin freely covered with a layer of its own fat-secretion thereby protected from invasion by the bacteria which always haunt the mouths of the epidermal glands?" "Have the fat people who get acne on a diet rich in fat less secretion on the surface of the skin to protect them against infection of the sebaceous glands?"

Subcutaneous accumulation of fat probably means a diminished excretion. Rosenfeld has made no researches wherewith as yet to solve these problems, but he suggests some interesting points bearing upon them.

"The Greeks of old, epicures in cleanliness and experts in the toilet, had as an adjunct to their baths a special process of rubbing the whole body with oil, probably to prevent the painful cracking which results from exposure to a dry climate and excessive loss of water by sweating. The Pustan shepherd[1] anoints himself with lard and hangs his shaggy sheepskin to windward; the Bushman curls himself up for his night's rest in a shallow pit well lined with raw hides, so that next morning, besmeared with fat and dust, he can start abroad protected for his wanderings under a brazen sky." From this Rosenfeld would argue that a covering of fat checks loss of water by perspiration and plays a considerable part in "heat-regulation."

Upon "**Heat-regulation in Universal Skin Diseases**" Linser[2] has just published some interesting observations in three cases of eczema. psoriasis, and erythrodermia exfoliativa (with pseudo-leukæmia). The difference between the surface temperature (taken by Oehler and Grünewald's method) and the rectal was found to be only about 1° C. instead of the $3° - 3\frac{1}{2}°$ in normal subjects. He suggests that dilatation of the capillaries of the skin in extensive disease may account for this approach of the external to the internal temperature. In such cases there is a greater quantity of blood exposed to the cooling influence of the air, consequently an increased "heat-loss" and greater "heat-production." This can be readily proved by placing the patient in a hot bath: in a few minutes the temperature rises in cases of wide-spread erythematous skin disease, while that of the normal person remains unchanged. So, too, in

[1] Pusta: the fertile plains bordering the Danube in Hungary.

[2] Linser, *Arch. f. Derm. u. Syph.*, 1906, lxxx. 249.

Linser's case of erythrodermia, a hot summer's day had the effect of sending up the temperature.

Now this increased heat-production indirectly argues an altered rate of metabolism, a problem which Linser set himself to study. The patient with **psoriasis** was placed on a mixed diet containing nitrogen 3,100 calories, or 50 calories per kilo of body weight. He was kept in bed, with sufficient covering to keep him warm in a room whose temperature was 15°—18° C. After five days the temperature was raised to 26°—30°, and the room sufficiently ventilated. Although the patient sweated freely, he did not feel uncomfortable and his appetite was good. The nitrogenous excretion was estimated by the Kjeldahl method, and the urea-N. by Krüger-Schmidt's.

A similar experiment was made in the case of **erythrodermia.**

In each case when the temperature of the room was raised (during a period of three days) the nitrogenous exchange was remarkably diminished, showing that the increased heat-loss caused by the skin disease very largely accounted for the high rate of exchange of energy.

In tabular form the results were as follows (averages per day):—

| Intake. | Output. | | Temperature of Room. |
|---|---|---|---|
| Food-Nitrogen. | Total N. (Urine and Fæces.) | Urea-N. | |
| *Psoriasis.* | | | |
| Gm. | | | |
| 25.3 | 25.7 | 0.75 | 16°—18° |
| 25.3 | 23.8 | 0.97 | 26°—30° |
| *Erythrodermia.* | | | |
| 27.9 | 27.2 | 1.04 | 16°—18° |
| 27.9 | 25.9 | 1.28 | 26°—30° |

The heat-production and heat-loss in these diseases is nearly double the normal. The clinical importance of this is, that such a rate of metabolism cannot be for long maintained without serious harm to the organism as a whole, and that if these extensive skin affections go uncured they will of themselves prove fatal in a year or two even apart from intercurrent disease.

The marked decrease in the rate of metabolism when the heat-loss was prevented by raising the temperature of the room to something approaching the normal body temperature is most instructive, and demonstrates that external warmth is a vital necessity in the treatment of these wide-spread skin affections that are accompanied by cutaneous hyperæmia.

Radaeli,[1] of Florence, has contributed a very important study

[1] Radaeli, *Arch. f. Dermat. u. Syph.*, 1906, lxxx. 323.

upon **the pathology of mycosis fungoides,** which adds not only to our knowledge of this disease in particular, but has a far wider application to disease in general. His paper, although by no means a dogmatic or final statement, is calculated to throw considerable light on a most debatable skin affection, and contains facts which, broadly interpreted, form a notable addition to pathology in its widest sense.

The condition Radaeli describes is one which Kaposi termed lymphodermia perniciosa, and is allied with pseudo-leukæmia cutanea on the one hand, sarcomatosis cutis on the other. His case was that of a man, aged 64, who eighteen months before death developed somewhat rapidly successive enlargement of all the lymphatic glands. A month or two after the first glands began to swell a skin eruption was observed—at first erythematous spots, which gradually developed into actual tumours. In spite of arsenic and anti-syphilitic treatment (the history of syphilis was almost certainly absent) the condition in no way improved, but progressed: the tumours in the skin increased and the glands enlarged everywhere, tonsils included; the liver and spleen grew to a size until the case presented the appearance of advanced lymphadenoma. The whole skin was a pale yellow, almost straw-colour, and œdematous except on the arms.

The eruption consisted of—(1) Pale, yellowish, erythematous spots, varying in size from a lentil to a halfpenny, in some cases covered with a fine branny scale; (2) Several round or oval papules, of a yellowish rosy-red colour, as large as a halfpenny or a penny; (3) Round nodules, from the size of a hazel-nut to an egg, rose-red, feeling fibrous or fleshy, situated subcataneously, with a smooth surface—in some places adherent to the skin and even ulcerated, and quite painless. At a late stage one set of glands in the left groin suppurated, and the abscess was opened, but neither in the pus nor in excised portions of skin, nor in the blood either during life or at the autopsy, could any micro-organisms be demonstrated.

There was at no time leucocytosis or abnormal differential count. The conditions found *post-mortem* briefly resolved themselves into a general infiltration of the skin with mononuclear lymphocytes, which by local aggregations gave rise to the tumours. These consisted almost entirely of lymphocytes with but scanty supporting stroma and blood-spaces lined by a single layer of endothelium. Here and there the lymphocytes were larger than usual, and contained several nuclei arranged peripherally (giant cells); the epidermis was thinned, the intercellular spaces widened, the cells of the rete malpighii cystic in places. Between the epidermis and the new infiltration tissue was a layer of œdematous corium and a few groups of tumour cells resembling in appearance and staining reactions mononuclear lymphocytes. There were some plasma cells and a few mast cells, but a complete absence of polynuclear cells betokening inflammation, no atypical cells associated with malignant growths and no hyperplasia of tissue.

On examining the glands, marrow, liver, spleen, and even kidneys (which were apparently in a state of acute parenchymatous nephritis), the same remarkable feature stood out saliently, that the enlargement of the organs depended, not upon the presence of any recognised inflammatory or neoplastic tissue, but upon a general invasion by these lymphocytes, slightly larger than normal and in great profusion.

Radaeli sums up excellently the observations of himself and other workers in this field. In this case the complete absence of any signs of an infective origin, of any "granulomatous" tissue, and of cells usually associated with lympho-sarcoma mark it as something apart. Plasma cells, mast cells, and giant cells are not commonly met with in sarcoma, nor mitotic figures in lymphocytes such as were found here. Moreover, the disease in the glands was the first change observed.

Unna had noticed in mycosis fungoides the polymorphism of the cells, the presence of a few plasma cells, abundant mast cells with frequent mitoses, and two forms of polynuclear cells—the one with irregularly-divided nuclei like myeloplaques; the other larger cells, the shape of whose nuclei and character of protoplasm was identical with the giant cells of tubercle or syphilis. Moreover, there were signs of protoplasmic disintegration in the presence of free albumin granules which in Radaeli's case were absent. He suggests that Unna's description relates to tumours undergoing spontaneous involution, while his own deals with the stage of active development. Philippson's description agrees in some details with each of the foregoing. He found, like Unna, polymorphism of cells, a few giant cells (in the early stage of the disease), and mitosis.

Most authorities agree that the primary focus of the neoplasm is in the blood. They are also agreed upon the occurrence of numerous small foci in the apparently sound skin.

Radaeli describes three stages in leukæmia and pseudoleukæmia:—

1. Enlargement of lymphatic organs without lymphocythæmia. (A-lymphomatous pseudo-leukæmia of Turk.)
2. Relative increase of lymphocytes without absolute leucocytosis. (Sub-lymphæmic lymphomatosis of Turk or lymphatic pseudo-leukæmia of Pinkus.)
3. Leukæmic stage, with enormous lymphocytosis. (Lymphatic leukæmia.)

The skin changes found in association with leukæmia and lymphadenoma are many and varied, while as to the exact diagnosis of mycosis fungoides there is the widest diversity of opinion. Radaeli ventures upon the suggestion that at least two groups of mycosis fungoides exist—cases associated with leukæmia or pseudo-leukæmia, and those other cases where the existence of leukæmia is not suggested. In the former class further variations may have to be recognised corresponding, perhaps, to the stages of leukæmia mentioned. For instance, Pelagatti reported in myelogenous leukæmia quite a different series of skin changes from those found by Radaeli in his case.

The conclusion finally propounded is that, whatever else the pathogeny may include, the disease arises in the blood, and depends in some way upon the vascular system for its dissemination. In fact, Radaeli inclines to the view that the truth lies somewhere between Ranvier and Gillot's old theory of "lymphadenie" of the skin and Kaposi's "lymphodermia perniciosa."

J. A. Nixon.

